# Pattern of Visits to Older Family Physicians in Taiwan

**DOI:** 10.3390/ijerph14050499

**Published:** 2017-05-08

**Authors:** Hao-Yen Liu, Cheng-Chieh Liu, Tzu-Hsiang Shen, Yi-Jen Wang, Jui-Yao Liu, Tzeng-Ji Chen, Li-Fang Chou, Shinn-Jang Hwang

**Affiliations:** 1 Department of Family Medicine, Taipei Veterans General Hospital, No. 201, Sec. 2, Shi-Pai Road, Taipei 112, Taiwan; yen.ee93@gmail.com (H.-Y.L.); jerryliu.lzj@gmail.com (C.-C.L.); jasper7593@gmail.com (T.-H.S.); sunnyclaire@hotmail.com (Y.-J.W.); tjchen@vghtpe.gov.tw (T.-J.C.); sjhwang@vghtpe.gov.tw (S.-J.H.); 2 School of Medicine, National Yang-Ming University, No. 155, Sec. 2, Linong Street, Taipei 112, Taiwan; 3 Department of Public Finance, National Chengchi University, No. 64, Sec. 2, Zhi-Nan Road, Taipei 116, Taiwan; lifang@nccu.edu.tw

**Keywords:** aged, ambulatory care, family physicians, national health programs, Taiwan

## Abstract

Many family physicians still practice at an old age. Nevertheless, their practice patterns have scarcely been studied. To address this lack of research, the current study analyzed claims data for a total of 2,018,440 visits to 171 family physicians in 2011 sourced from Taiwan’s National Health Insurance Research Database. Family physicians aged 65 years and over had fewer patients (mean: 2330, standard deviation (SD): 2019) and visits (mean: 9220, SD: 8600) than younger physicians had. Furthermore, the average age of the patients who visited physicians aged 65 years and over was 51.9 (SD: 21.5) years, significantly higher than that of patients who visited younger physicians. However, the proportions of visits for upper respiratory tract infections, hypertension, diabetes mellitus, and dyslipidemia did not differ significantly among different age groups of physicians. In the future, the manpower planning of physicians should take into consideration the age structure and work profile of physicians.

## 1. Introduction

In most countries, physicians have no statutory retirement age [1,2,3]. According to the 2015 State Physician Workforce Data, 28.4 percent of actively licensed physicians in 2014 in USA were 60 years of age or older [4]. Although clinical performance usually improves with experience [5], an expected age-related decline in motor skills and cognitive abilities is still inevitable among older physicians [6,7,8,9,10]. In health policy discussions regarding the physician workforce, an older physician is typically not regarded as equivalent to a younger colleague because an older physician will typically work fewer hours, have a lighter workload, and have service content that is less intensive [11,12,13]. However, while most studies of this subject have focused on more experienced internists [7] and senior surgeons involved in complex procedures [8,14], few have dealt with older family physicians, especially on a nationwide scale.

In Taiwan, family medicine (FM) is the main specialty that provides primary care services including minor illnesses, preventive healthcare, health checkups, and referral for major disease in the community [15]. In addition, some health services such as child preventive care and cancer screening are optional for family physicians. Under the National Health Insurance (NHI) system, patients are able to choose or change their healthcare providers freely, and physicians are paid by the fee-for-service payment system [15,16]. The aim of our study was to compare the practice patterns of FM physicians of different age groups. The points of comparison included service volume, service content, and the patients’ age distribution. The findings may serve to make policymaking regarding the healthcare workforce and its training better informed.

## 2. Materials and Methods

### 2.1. Data Collection

Taiwan’s NHI program was initiated in 1995 and provides healthcare to 99.6% of Taiwan’s residents. For this study, we obtained datasets from the related National Health Insurance Research Database, which contains complete NHI claims data in an electronic form. Specifically, the datasets used in this study included the claims submitted by 10% of all physicians in 2011. These physicians were randomly sampled from all the physicians contracted by the NHI program. There were a total of 32,801,069 ambulatory records associated with these physicians. Each record contained fields indicating the healthcare facility, visit date, specialty, the patient’s identification number and birthdate, up to three diagnoses, and the physician’s identification number. The diagnoses were coded according to the International Classification of Diseases, 9th Revision, Clinical Modification (ICD-9-CM). Additionally, we used the master data file for healthcare facilities (HOSB2011.DAT) to identify the accreditation level and location of a given healthcare facility, and the master data files regarding specialists (DOC2011.DAT) and healthcare personnel (PER2011.DAT) in order to determine the age and sex of each board-certified physician.

The institutional review board of Taipei Veterans General Hospital approved the study (2013-10-001CE).

### 2.2. Study Design

From the datasets, we extracted the ambulatory claims belonging to board-certified FM physicians working at primary care clinics in 2011. That is, we excluded claims records for any physicians without at least 365 visits in the year because we classified them as part-time workers. We further excluded any trainees and those physicians practicing in the outpatient departments of hospitals. As a result, this study included claims data for a total of 171 FM physicians.

These physicians were divided into three age groups: <50 years old, 50–64 years old, and ≥65 years old. Each physician’s practice location was categorized as urban, suburban, or rural according to the urbanization stratification of Taiwan townships developed by Taiwan’s National Health Research Institutes [17]. Of the 7 urbanization levels in that stratification, the first and second were deemed urban, the third and fourth were deemed suburban, and the fifth to seventh plus the isolated islands of Taiwan were regarded as rural.

For each FM physician, we calculated the total numbers of patients and visits in 2011. Among the visits, we also calculated the percentages of visits regarding selected acute and chronic illnesses. For acute illnesses, we selected upper respiratory illnesses (URI) (ICD-9-CM: 460-466) as the principal diagnosis, and for chronic illnesses, we selected diagnoses of hypertension (ICD-9-CM: 401-405), diabetes mellitus (ICD-9-CM: 250) and dyslipidemia (ICD-9-CM: 272). Furthermore, we computed the average age of patients counted by patient-visits and distribution of patient ages across all the visits.

### 2.3. Statistical Analysis

We used the programming software Perl version 5.20.2 (https://www.perl.org/) for data processing and the statistical software R version 3.3.2 (https://www.r-project.org/) for statistical analysis. The Kruskal-Wallis test was performed to examine the difference between three age groups. Statistical significance was determined as a *p*-value of ≤0.05. If there were difference across the three groups, we proceed to further analysis with Wilcoxon–Mann–Whitney test to examine two group comparisons. After a Bonferroni correction applied for multiple comparisons, a *p*-value < 0.0167 was considered statistically significant. A boxplot was constructed to present the age distribution for all the patients seen by each physician, with the resulting boxplots then stratified by the physicians’ age groups. For each boxplot of a physician, the bottom of the box indicated the 25th percentile, the top of the box indicated the 75th percentile, and the line in the middle indicated the 50th percentile of the patients’ age distribution across all the visits to that physician.

## 3. Results

### 3.1. Physicians’ Characteristics

Of the 171 active FM physicians with a mean age of 53.6 ± 12.2 years, 90.6% (n = 155) were male and 53.2% (n = 91) practiced in urban areas (Table 1). While 19.4% of the male physicians were aged ≥65 years, there were no practicing female physicians aged ≥65. According to the Taiwan Medical Association (TMA) report in 2011, the proportion of total male physicians in Taiwan was 83.7%, and female physicians aged ≥65 accounted for 0.2% of all physicians [18]. The distributions of practice locations were similar among the three age groups.

### 3.2. Patients, Visits, and Disease Patterns

In total, the 171 FM physicians had 2,018,440 visits in the year. The physicians aged ≥65 years had significantly fewer patients than the younger physicians (Table 2). The physicians aged ≥65 years also had fewer visits, but the difference in the number of visits from those of physicians aged <50 years was statistically insignificant after further analysis (*p* = 0.289).

Of all visits made to the 171 FM physicians, 36.7% (n = 740,886) involved URI as the patient’s principal diagnosis. The shares of URI-related visits did not differ significantly among the three age groups of physicians (Table 2). Otherwise, 11.5% (n = 231,806) of all the visits involved hypertension, diabetes mellitus, or dyslipidemia as the principal diagnosis, and the shares of visits for these diagnoses also did not differ significantly among the three age groups of physicians.

### 3.3. Patients’ Age Distribution of Visits

The average age of the patients counted by patient-visits to physicians aged ≥65 years was 51.9 ± 21.5 years, significantly higher than those of the patients who made visits to physicians aged 50–64 years (47.4 ± 22.9, *p* < 0.001) and physicians aged <50 years (43.2 ± 24.8, *p* < 0.001).

**Figure 1:**
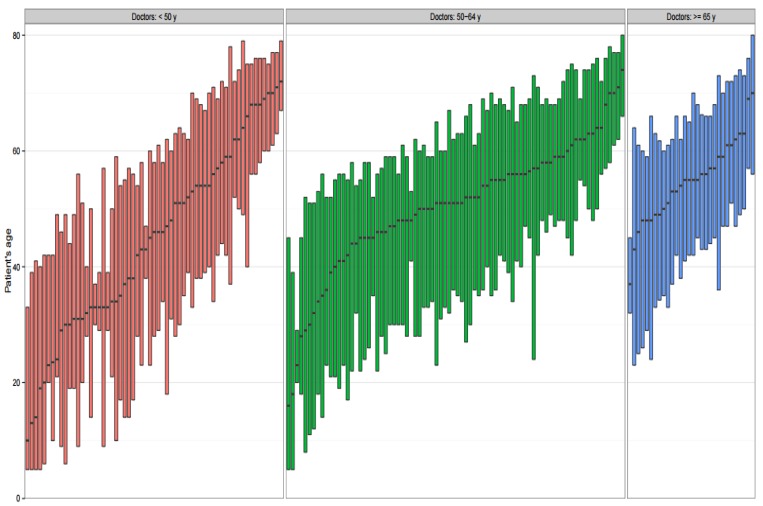
Patients’ age distribution for visits to each physician (sorted according to median age for each physician age group). Each red, green, and blue boxplot represented the physician age groups <50 years old, 50–64 years old, and ≥65 years old, respectively.

We demonstrated the patients’ age distribution for visits to each physician with a boxplot and then arranged all the boxplots according to the median age for the three physician age groups (Figure 1). Physicians aged ≥65 years had remarkably fewer pediatric patient visits and higher proportions of visits by middle-aged and elderly patients.

## 4. Discussion

This study found that older FM physicians had fewer patients and outpatient visits than younger physicians. In Taiwan, the proportion of physicians older than 60 years increased from 13% in 2000 to 18% in 2015, according to a 2015 report from the TMA [19]. More recently, a 2016 study based on AMA Physician Masterfile data reported that primary physicians tend to retire in their mid-60s [20]. Unlike with professions related to public safety, such as pilots [21], the establishment of a mandatory retirement age for physicians remains controversial in most countries [22,23,24]. The factors influencing a senior physician’s decision to retire from clinical practice often include workload, health, and career satisfaction [24,25,26]. Accordingly, during the transition period from working to retirement, physicians may try to adjust their work pattern in various ways, perhaps by taking up more clinical teaching work [27,28] or by gradually reducing their working hours to decrease their clinical workload [2,24,28]. Our finding that physicians aged ≥65 years had fewer patients and visits than younger physicians may suggest changes in practice patterns, such as decreasing productivity, shifting to other tasks, or economical considerations in late career as transition into retirement begins. However, we found no significant difference between the number of visits to physicians aged ≥65 years and the number of visits to physicians aged <50 years, possibly because older physicians have more previous visits or established clientele and thus a higher rate of returning patients than younger physicians. Further studies are required to clarify whether patients visiting older physicians have more loyalty.

It is notable that we found similar proportions of visits for acute and chronic illnesses for each age group of physicians. This finding might suggest that there were no differences in the prevalence rates of common diseases among patients seen by older and younger physicians. Our data revealed that acute illness accounted for over 30% of all illness-related visits and was twice as frequent for chronic illness, which is consistent with a previous study report, wherein FM physicians were found to spend more time in visits of acute care than visits of chronic care [29]. Chronic illnesses including hypertension, diabetes mellitus, and dyslipidemia constituted less than half of all acute illnesses. Family physicians might spend more time on visits when dealing with a chronic illness, especially for compliance assessments, negotiations, and the provision of nutrition advice [30]. In the U.S. in 2015, family physicians had an average of 74 office patient encounters per week over a practice average of 47 weeks [31]. However, according to our study, FM physicians in Taiwan have about three times more outpatient visits, possibly because of the easy accessibility and affordable co-payment system under the NHI system [15]. Simply put, large numbers of patients waiting in a clinic cause physicians to be extraordinarily busy and require them to keep visits relatively short, a situation which inevitably causes physicians to pay less attention to chronic illness patients [32,33].

Our results support the view that older FM physicians are less likely to provide care for pediatric patients than younger FM physicians [34]. In addition, the mean age of patients who visited physicians aged ≥65 years was significantly higher than that of patients who visited younger physicians. There were three potential explanations for this finding. First, the younger physicians were more strongly urged to provide a broad range of clinical services, including as pediatric preventive services, in order to establish their clientele. Relatedly, one study reported that less experienced physicians had more costly practice patterns because they treated more complex, high-risk patients [35]. Second, previous studies have shown that physicians have weak abilities to accurately self-assess and limited competence in perceiving their own cognitive declines [14,36]. This phenomenon might be observed in older physicians who could not provide a broad range of services due to a lack of self-directed learning. Third, patients might be more loyal to those physicians they have had a long-term relationship with.

There were some limitations to our study. First, although URI accounted for most of the acute illness diagnoses, some visits relating to other diagnoses, such as gastritis or other soft tissue diseases, may have been neglected in our study. Second, we used the number of patients and visits as parameter to evaluate the service volume of FM physicians. However, there were differences in the average physician–patient encounter time for different circumstances such as new visits or follow-up visits for each FM physician [37]. Further studies using parameters such as total patient contact hours per week would thus be helpful in better understanding the workloads of FM physicians.

## 5. Conclusions

In Taiwan in 2011, family physicians aged 65 years and over accounted for one fifth of active practicing family physicians. Older family physicians tended to have fewer patients than younger physicians did. However, the proportions of visits for upper respiratory tract infections and for hypertension, diabetes mellitus, and dyslipidemia did not differ significantly among the different age groups of physicians. In the future, the manpower planning of physicians should take into consideration the age structure and work profile of physicians.

## Figures and Tables

**Table 1:** Family physicians’ characteristics by age groups.

	Age Group			Total
	<50 Years Old	50–64 Years Old	≥65 Years Old	
Number, n (%)	61 (35.6)	80 (46.8)	30 (17.5)	171
Median age (years old)	41	58	69	55
Male, n (%)	51 (83.6)	74 (92.5)	30 (100)	155 (90.6)
Practice location, n (%)				
Urban	32 (52.4)	44 (55.0)	15 (50.0)	91 (53.2)
Suburban	21 (34.4)	22 (27.5)	10 (33.3)	53 (30.9)
Rural	8 (13.1)	14 (17.5)	5 (16.7)	27 (15.7)

**Table 2:** Patients, visits and disease patterns for different age groups of family physicians.

	Age Group			Kruskal–Wallis Test	Wilcoxon–Mann–Whitney Test
	<50	50–64	≥65	*p*-Value	*p*-Value
Patients, n				0.006	
Mean	4058	3855	2330		0.012 (<50 vs. ≥65)
SD	3073	2474	2019		<0.001 (50–64 vs. ≥65)
Visits per year, n				0.021	
Mean	11,019	13,371	9220		0.289 (<50 vs. ≥65)
SD	8837	8992	8600		0.006 (50–64 vs. ≥65)
Proportion of acute illness visits				0.532	
Mean	0.369	0.347	0.314		
SD	0.228	0.215	0.199		
Proportion of chronic illness visits				0.429	
Mean	0.152	0.154	0.11		
SD	0.163	0.17	0.122		
